# Case Report: Ipilimumab-Induced Panhypophysitis: An Infrequent Occurrence and Literature Review

**DOI:** 10.3389/fonc.2020.582394

**Published:** 2020-12-01

**Authors:** Agnese Barnabei, Silvia Carpano, Alfonsina Chiefari, Marta Bianchini, Rosa Lauretta, Marilda Mormando, Guilia Puliani, Giancarlo Paoletti, Marialuisa Appetecchia, Francesco Torino

**Affiliations:** ^1^ Oncological Endocrinology Unit, IRCCS Regina Elena National Cancer Institute, Rome, Italy; ^2^ Division of Medical Oncology 2, IRCCS Regina Elena National Cancer Institute, Rome, Italy; ^3^ Department of Systems Medicine, Medical Oncology, University of Rome Tor Vergata, Rome, Italy

**Keywords:** immune checkpoint inhibitors, ipilimumab, hypophysitis, panhypophysitis, diabetes insipidus

## Abstract

**Background:**

Immune checkpoint inhibitors (ICIs), by unleashing the anticancer response of the immune system, can improve survival of patients affected by several malignancies, but may trigger a broad spectrum of adverse events, including autoimmune hypophysitis. ICI-related hypophysitis mainly manifests with anterior hypopituitarism, while the simultaneous involvement of both anterior and posterior pituitary (i.e., panhypophysitis) has rarely been described.

**Case Presentation:**

In June 2015, a 64-year-old man affected by liver metastases of a uveal melanoma was referred to us due to polyuria and polydipsia. Two months prior, he had started ipilimumab therapy (3 mg/kg iv every 21 days). The treatment was well-tolerated (only mild asthenia and diarrhea were reported). A few days before the fourth cycle, the patient complained of intense headaches, profound fatigue, nocturia, polyuria (up to 10 L urine/daily), and polydipsia. Laboratory tests were consistent with adrenal insufficiency, hypothyroidism, and transient central diabetes insipidus. The pituitary MRI showed an enlarged gland with microinfarcts, while the hypophyseal stalk was normal, and the neurohypophyseal ‘bright signal’ in T1 sequences was not detected. The treatment included dexamethasone (then cortisone acetate at replacement dose), desmopressin, and levothyroxine. Within the next five days, the symptoms resolved, and blood pressure, electrolytes, glucose, and urinalysis were stable within the normal ranges; desmopressin was discontinued while cortisone acetate and levothyroxine were maintained. The fourth ipilimumab dose was entirely administered in the absence of further side effects.

**Conclusion:**

As ICIs are increasingly used as anticancer agents, the damage to anterior and/or posterior pituitary can be progressively encountered by oncologists and endocrinologists in their clinical practice. Patients on ICIs and their caregivers should be informed about that risk and be empowered to alert the referring specialists early, at the onset of panhypopituitarism symptoms, including polyuria/polydipsia.

## Introduction

Immune checkpoint molecules are essential in the negative modulation of the immune response, but also play a key role in cancer cell immune escape (1). Immune checkpoint inhibitors (ICIs) are monoclonal antibodies (mAb) that, by targeting specific immune checkpoints (e.g., the Cytotoxic T-Lymphocyte Antigen-4 receptor, CTLA-4; the programmed death receptor, PD; and its ligand, PD-L1), unleash the anticancer immune response blocked by the cancer itself. In recent years, ICIs have shown durable antitumor responses and improved survival in patients affected by a broad spectrum of malignancies (2).

In 2011 Ipilimumab, an ICI targeting the CTLA-4 receptor, was the first ICI approved for the treatment of patients with advanced malignancy, namely advanced melanoma. Recently, ipilimumab received FDA approval as adjuvant treatment in patients affected by high-risk non-metastatic melanoma (2).

ICIs, due to their mechanism of action, may trigger immune-related adverse events (irAEs), mainly involving skin (rash), gastrointestinal tract (diarrhea and colitis), liver (elevated transaminases), and endocrine system. In the context of ICI-related endocrine toxicity, the incidence of hypophysitis was higher in patients under ipilimumab–nivolumab combination therapy (6.4%) compared with those under anti-CTLA-4 therapy (3.2%), anti-PD-1 therapy (0.4%), and anti-PD-L1 therapy (<0.1%) (3).

ICI-induced hypophysitis mainly affects the anterior pituitary, being TSH, ACTH, and FSH/LH secreting cells that were more frequently damaged. The damage of the posterior pituitary by ICIs, manifesting with central diabetes insipidus (CDI) is anecdotic (3).

Herein, we report the case of a patient diagnosed with simultaneous anterior and posterior hypophysitis (panhypophysitis) induced by ipilimumab and discuss the emerging difference in the incidence of hypophysitis/CDI among subclasses of ICIs and the related pathogenic mechanisms.

## Case Presentation

In June 2015, a 64-year-old man was referred to us due to polyuria and polydipsia, in the absence of a medical and family history of endocrine disorders and autoimmune diseases. In 2004, he underwent ocular proton beam radiotherapy for a left eye uveal melanoma, obtaining the complete disease remission. In February 2015, liver metastases were documented, and the pathology evaluation of biopsy samples showed the infiltration of wild type BRAF melanoma cells. Therefore, in March 2015, the patient started ipilimumab therapy (3 mg/kg iv every 21 days). The treatment was well-tolerated, as only grade 1 asthenia and diarrhea were reported. A few days before the fourth cycle, the patient complained of intense headaches, profound fatigue, nocturia, polyuria (up to 10 L urine daily), and polydipsia. Neither visual impairment nor diplopia were reported. Laboratory tests (**Table 1**) were consistent with the diagnosis of central adrenal insufficiency, hypothyroidism, and transient diabetes insipidus.

**Table 1:** Patient biochemical parameters.

Biochemical Parameters	Values at the diagnosis	Values before the diagnosis	Normal Range
Plasma osmolarity (mOsm/kg)	314	NA	275–295
Urine osmolarity (mOsm/kg)	174	NA	400–1,100
TSH (mcU/ml)	0.06	2.1	0.5–3.5
FT3 (pg/ml)	2.3	3.0	2–4.4
FT4 (pg/ml)	5.2	14.2	9.3–17
ACTH (pg/ml)	1	22.7	7.2–63.3
Blood cortisol (mcg/l)	2.6	132	62–194
Testosterone (ng/ml)	2	4.9	3–7
FSH (mUI/ml)	5	11.6	1.3–19.3
LH (mUI/ml)	3	8.9	1.8–12
IGF-1 (ng/ml)	97	NA	64–188
Glycemia (mg/dl)	133	110	60–110
Na^+^ (mEq/l)	139	141	135–148
K^+^ (mEq/l)	5.2	4.1	3.5–5.1
Creatinine (mg/dl)	1.06	1.0	0.67–1.17
Urea (mg/dl)	53	48	15–50

**Figure 1:**
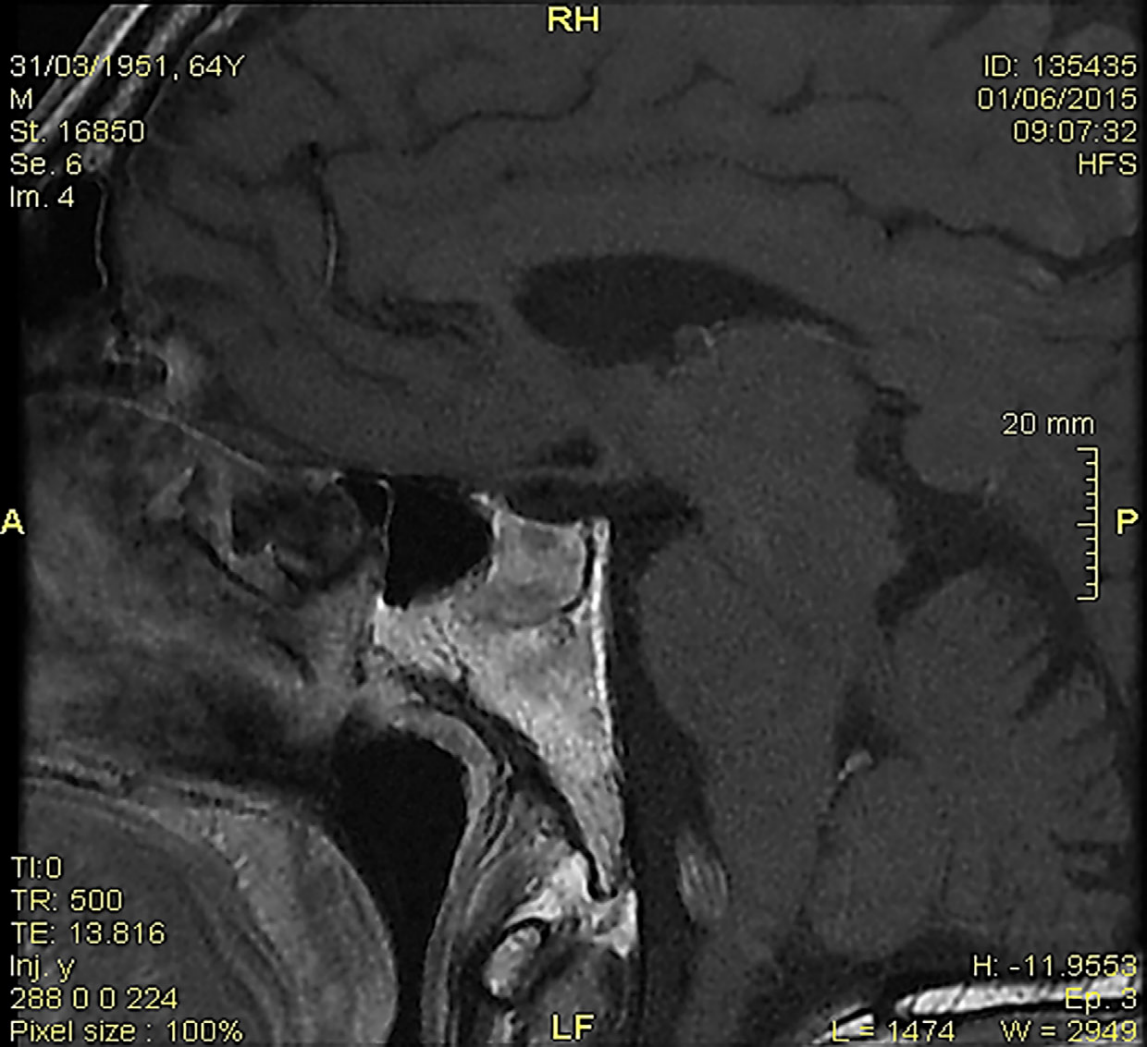
At the magnetic resonance imaging, pituitary microinfarcts in an enlarged gland were shown; the hypophyseal stalk was normal, and the neurohypophyseal ‘bright signal’ in T1 sequences was undetectable.

The brain magnetic resonance imaging (MRI) showed pituitary microinfarcts in an enlarged gland, while the hypophyseal stalk was normal, and the neurohypophyseal ‘bright signal’ in T1 sequences was not detected (**Figure 1**). As the patient refused to be hospitalized, he was prescribed home drug therapy: dexamethasone 4 mg IM every 6 h for four days, progressively tapered down and switched to cortisone acetate at replacement dose; desmopressin 60 mg tablets (modifiable according to polyuria and polydipsia); levothyroxine 50 mcg/days. The symptoms progressively resolved within five days. Desmopressin was discontinued, maintaining cortisone acetate and levothyroxine. Blood pressure, electrolytes, glucose, and urinalysis were stable within the normal ranges.

As the patient completely recovered, the fourth ipilimumab dose was entirely administered with a delay of 23 days. No further side effects were reported. The malignancy remained stable for two years. In June 2017, as liver metastases progressed, nivolumab was administered. The treatment was well tolerated with mild skin and gastrointestinal toxicity, and it was continued up to July 2018, when the patient’s clinical conditions worsened, and progressive disease was shown. In August 2018, the patient died due to hepatic failure.

## Discussion

Hypophysitis is classified as adenohypophysitis, infundibulo-neuro-hypophysitis, or panhypophysitis when the anterior lobe, the posterior lobe, and the stalk of the gland, or both, are damaged, respectively (4). Based on pathology, two common forms of hypophysitis (lymphocytic and granulomatous) and three rarer variants (xanthomatous, necrotizing, and plasma-cell rich) are documented (4). The etiological classification identifies primary and secondary forms. Primary hypophysitis, the most common form, has autoimmune pathogenesis with no obvious causative agent (4). Secondary hypophysitis includes local and systemic disease and treatment-related hypophysitis. For local disorders, inflammation of the pituitary appears as a reaction to a sellar disease (i.e., Rathke’s cleft cyst, craniopharyngioma, germinoma, and pituitary adenoma). For systemic diseases, hypophysitis stems from the involvement of different organs by infectious or inflammatory disorders (e.g., Wegener’s granulomatosis, sarcoidosis, tuberculosis, syphilis, or sellar metastases). Treatment-related hypophysitis may derive from complications of surgery and/or radiotherapy or as a side effect of certain drugs (4).

Patients with anterior hypophysitis usually present nonspecific symptoms, including headache, visual impairment, fatigue, weakness, confusion, memory loss, erectile dysfunction and loss of libido, anorexia, labile moods, insomnia, temperature intolerance, subjective sensation of fever, and chills (4). MRI is the imaging modality of choice in the differential diagnosis of pituitary diseases, including hypophysitis (4–6). In ICI-induced hypophysitis, MRI may show a diffuse enlargement of the pituitary, irregular thickening of the infundibulum, and diffuse enhancement (4–6). However, approximately 23% of cases may have a normal MRI despite clinical evidence of ICI-induced hypophysitis (4). Notably, the entity and quality of pituitary damage triggered by ICIs may depend on the time when an MRI assessment is made in respect of the onset of symptoms and/or the treatment start. Levels of ACTH, cortisol, TSH, and/or free T4, GH, prolactin, insulin-like growth factor I (IGF-1), follicle-stimulating hormone (FSH), luteinizing hormone (LH), and testosterone are variably altered, indicating different degrees of hypopituitarism (4).

CDI results from a deficiency of vasopressin (antidiuretic hormone, ADH) due to a hypothalamic–pituitary disorder. Most CDI cases are idiopathic or result from primary or secondary cancers, or infiltrative diseases (i.e., Langerhans cell histiocytosis) (7). Less frequently, CDI may be caused by familial and congenital disorders, neurosurgery or trauma, hypoxic encephalopathy, post-supraventricular tachycardia, and anorexia nervosa (7, 8). Drugs may rarely trigger CDI, including lithium, amphotericin-b, cidofovir, demeclocycline, didanosine, foscarnet, ofloxacin, orlistat (5). Patients with CDI typically present polyuria, nocturia, and, due to the initial elevation in serum sodium and osmolality, polydipsia. In the case of underlying neurologic diseases, neurologic symptoms may be present as well (8). The serum sodium concentration in untreated CDI is often in the high normal range, which stimulates thirst to replace the urinary water loss. Moderate to severe hypernatremia can develop when thirst is impaired, or autonomous drinking is hampered (8). Clinical features of CDI may differ based on whether disorders act at one or more of the sites involved in ADH secretion: the hypothalamic osmoreceptors; the supraoptic or paraventricular nuclei; or the superior portion of the supra-opticohypophyseal tract (7, 8). When the damage hits the tract below the median eminence or the posterior pituitary, usually causes only transient polyuria, because ADH produced in the hypothalamus can still be secreted into the systemic circulation *via* the portal capillaries in the median eminence (7). In persistent CDI, MRI high signal intensity of the posterior pituitary may be absent as a result of failure to synthesize, transport, or store ADH granules, being the pituitary gland and pituitary stalk homogeneously enhanced following the administration of contrast enhancement (9).

The diagnosis of CDI is based on the polyuria–polydipsia syndrome, on serum/plasma and urine studies, and the response to exogenous vasopressin, being an absent response suggesting nephrogenic diabetes insipidus. A water deprivation test (showing failure to concentrate urine maximally) or ADH serum levels may be required to confirm the clinical diagnosis of CDI. Recently, copeptin is proposed to improve the accuracy of CDI diagnosis. CDI treatment requires desmopressin or lypressin (10).

Panhypopituitarism derives from the damage to the anterior hypophysis and either one of the sites of ADH production (the supraoptic or paraventricular nuclei; or the superior portion of the supra-opticohypophyseal tract) or of storage (hypophyseal stalk and neurohypophysis). In patients with panhypopituitarism, symptoms of CDI enrich the syndrome of anterior hypophysitis. CDI is also present in 17–48% of patients diagnosed with an autoimmune hypophysitis (11).

In the past, drug-induced hypophysitis was rarely diagnosed, mainly in patients under interferon or ribavirin (12). Recently, with the advent of ICIs, hypophysitis is among the most diagnosed endocrine irAEs induced by those agents, resulting in hypopituitarism leading to central adrenal insufficiency, central hypothyroidism, and hypogonadotropic hypogonadism. GH and prolactin abnormal levels occur less frequently. The incidence of ICI-induced hypophysitis is reported between 4–20% with ipilimumab, 1,8% with tremelimumab, 0.6% with nivolumab, and 0.7% with pembrolizumab, 8% with the combination ipilimumab plus nivolumab or pembrolizumab (10). ICI-hypophysitis is reported 2–5 times more frequently in men (particularly in over 60 old ones) than in women: that is the opposite of the other forms of hypophysitis (13).

In a very recent study based on the WHO global database of individual case safety reports, Bai et al. (14) showed that, in the period from January 2011 to March 2019, a total of 6,089 ICI-related endocrine AEs were reported, of which 1,144 (18.8%) were pituitary events, including hypophysitis, hypopituitarism, and pituitary enlargement. The anti-CTLA-4 subgroup had a stronger association with hypophysitis/hypopituitarism than the anti-PD (anti-PD-1/anti-PD-L1) subgroup. Among ICI-associated hypophysitis/hypopituitarism cases, the proportion of males was higher than females (630 [63.9%] vs. 356 [36.1%]). Anti-CTLA-4 subgroup and ICI combination (ipilimumab–nivolumab) subgroup both had a significantly earlier onset time than anti-PD subgroup (67 days [48–87]; 90 [34–155]; 140 [62–218], both *p <*0.05). CDI was reported in seven out of 1,072 (0.7%) of the registered hypophysitis/hypopituitarism cases among patients who received an ICI as a single agent or in combination (ipilimumab–nivolumab) (14).

High-dose corticosteroids were recommended for the treatment of ipilimumab-induced hypophysitis (15, 16). However, their use remains controversial as it lacks high level evidence (3). High-dose corticosteroids are currently recommended for patients with ICI-induced hypophysitis when severe hyponatremia or significant mass effect from pituitary swelling are shown, in the grade 3–4 endocrine irAEs, including thyroid storm and Graves’ ophthalmopathy, or in the setting of critical illness (3, 17, 18). In the other cases, corticosteroids at replacement dose are used, together with other replacement treatment(s) according to the emerged hormone deficit(s) (17).

As a result of searching the available literature, CDI was rarely reported as either in a panhypophysitis syndrome or as a single endocrine ICI-related adverse event. To the best of our knowledge, only three cases of panhypophysitis induced by ipilimumab are available in the literature: in two of them, ipilimumab was administered as a single agent (19, 20), while in the third case it was combined with nivolumab (an anti-PD-1 mAb) (21). The first patient was affected by prostate cancer, the other two were affected by cutaneous melanoma (19–21). More recently, three case reports of isolated ICI-related CDI were published. In the first case, CDI occurred in a patient affected by Merkel cell carcinoma who received avelumab (an anti-PD-L1 mAb) (22). In the second one, CDI was reported in a patient affected by lung cancer who received nivolumab (an anti-PD-1 mAb) (23), while in the third case, CDI was diagnosed in a patient affected by mesothelioma, who was treated with an experimental combination of tremelimumab (an anti-CTLA4 mAb) and durvalumab (an anti-PD-L1 mAb) (24).

The pathogenesis of ICI-related damages to anterior hypophysitis is still unclear. Multiple contributing mechanisms were suggested, including: a) the prevalent expression of CTLA-4 antigens on some pituitary cells; b) the prevalent intensity of types II and IV hypersensitivity immune reaction triggered by different ICI-subclasses; (3) the role of CTLA-4 gene polymorphisms.

CTLA-4 antigens were found predominantly expressed in thyrotroph and lactotroph pituitary cells both in mice and in humans (25). Iwama et al. (26) obtained the first murine model of anti-CTLA-4-related (anterior) hypophysitis, following repeated administrations of an anti-CTLA4 mAb. They not only demonstrated pituitary infiltration with hematopoietic mononuclear cells (mainly CD45+ lymphocytes) but also production of serum antibodies directed against the anterior pituitary cells, acting through complement-mediated cell cytotoxicity. Recently, anti-pituitary autoantibodies, mainly against pituitary cells secreting TSH, FSH, and ACTH, were detected in patients with ipilimumab-induced hypophysitis (27). In specimens from the pituitary of patients who received tremelimumab, an anti-CTLA-4 IgG2 mAb, the expression of CTLA-4 was found to be increased, and the clinical-pathological features were compatible with type II and type IV hypersensitivity reactions (27).

Interestingly, IgG subclasses demonstrated different potency in activating ADCC and the classical complement pathway, with IgG1 exerting the relatively more potent effects compared with IgG2 and IgG4 subclasses (28). Moreover, IgG4 are unable to activate the classical complement pathway (28). Among anti-CTLA4 mAbs, ipilimumab is an IgG1 mAb, while tremelimumab is an IgG2 mAb. Among anti-PD1/PD-1L mAbs, avelumab and durvalumab are IgG1 mAbs, while nivolumab and pembrolizumab are IgG4 mAbs (3). All the above factors may explain the occurrence of hypophysitis in patients under CTLA4-mAbs and the higher incidence of hypophysitis in patients on treatment with ipilimumab (IgG1) compared with anti-PD1/PD-L1 mAbs. These differences in drug structure may provide a further causative explanation for the different rates of pituitary toxicity among ICIs. However, the pathogenic contribution of each single mechanism in triggering ICI-related hypophysitis remains to be investigated (3, 4, 12).

Finally, a potential role of some gene polymorphisms in prompting ICI-related toxicity has been envisioned (27). Some *CTLA-4* polymorphisms are commonly associated with various autoimmune disorders, including endocrinopathies (i.e., type 1 diabetes mellitus, Graves’ disease, autoimmune hypothyroidism, and Addison disease) (29). Only in few studies a linkage of *PD-1* polymorphisms with T1DM was suggested, while *PD-L1* polymorphisms was found to be linked to type 1 diabetes mellitus, Graves’ disease, and Addison disease (3). No reports have linked *PD-L2* polymorphisms to autoimmune endocrinopathies (3). Based on these data and on the well documented association between some germline genetic variants and adverse events induced by anticancer agents (30), an association between some immune checkpoint gene polymorphisms and the development of ICI-induced toxicities has been postulated (31). However, only very few studies evaluated the association between polymorphisms of immune checkpoint genes and irAEs, including immune-related endocrinopathy (32, 33). In those studies, no certain causative link was found between polymorphisms of immune checkpoint genes and toxicities induced by ICIs (3).

If the pathogenesis of anterior hypophysitis remains to be fully understood, the lack of experimental and pathology findings makes it even harder suggesting pathogenic hypotheses for the ICI-related CDI occurrence, both as ICI-related panhypophysitis and as single endocrine irAE.

In a simplifying framework, the posterior pituitary damage would theoretically derive from the extension of the ICI-induced autoimmune inflammation of the anterior pituitary to the posterior gland. On the other hand, panhypophysitis would stem from factors triggering the concurrent damage to both the anterior pituitary and to one of the anatomical sites of the hypothalamus–hypophysis axis.

Unfortunately, imaging techniques, such as MRIs, may not help diagnose ICI-related CDI/hypophysitis, since pituitary MRI may be normal in patients diagnosed with hypophysitis and/or CDI (4–9).

Importantly, pathology data would be of great value in clarifying the pathogenic mechanisms of ICI-related panhypophysitis. Unfortunately, the solely available autopsy report describes findings of an anterior hypophysitis, in the absence of aspects suggesting any involvement of posterior pituitary.

In parallel, the pathogenesis of ICI-induced selective damage to posterior pituitary manifesting as isolated CDI remains cryptic. An autoimmune reaction triggered by ICIs would suggest the selective hit to posterior pituitary and/or supraoptic and paraventricular nuclei. To this aim, the assessment of antibodies to ADH-secreting cells of the human hypothalamus (AHA) could be useful, as they are in the CDI diagnosis (3, 23). However, when available, the serum assessment of anti-nuclear antibodies, extractable nuclear antigen, and anti-neutrophil cytoplasmic antibodies, may not reveal the presence of an autoimmune disease (3, 23). Unfortunately, the current lack of clinic-pathological evidence renders those hypotheses merely speculative.

## Conclusion

The occurrence of panhypopituitarism, despite its rarity, should be considered in patients who are treated with ipilimumab, both as a single agent or in ICI-combination. Moreover, recent reports highlight that isolated CDI may occur in patients under anti-PD1/PD-L1 mAbs.

Due to the increasing clinical use of ICIs as anticancer agents in several malignancies, not only in the metastatic, but also in the adjuvant clinical setting, the consequences of ICI-induced damage to either anterior or posterior pituitary or both, can be encountered by oncologists and endocrinologists in current clinical practice. Patients on ICIs and their caregivers should be informed about the risk of ICI-induced hypophysitis and empowered to alert referring health care professionals early, at the onset of symptoms of panhypopituitarism, including polyuria/polydipsia.

## Data Availability Statement

The original contributions presented in the study are included in the article/supplementary material, further inquiries can be directed to the corresponding author.

## Ethics Statement

The written informed consent was obtained from the patient for the publication of any potentially identifiable images or data.

## Author Contributions

FT, AB, and MA wrote the manuscript. SC, AC, MB, RL, MM, and GP collected the data. AB, FT, MA, SC, and GP contributed to the project development. All authors contributed to the article and approved the submitted version.

## Conflict of Interest

The authors declare that the research was conducted in the absence of any commercial or financial relationships that could be construed as a potential conflict of interest.
